# Research on Integral Fabrication and Inner Surface Metallization of the High-Frequency Terahertz Hollow-Core Metal Rectangular Waveguide Cavity by a Combined Process Based on Wire Electrochemical Micromachining and Electrochemical Deposition

**DOI:** 10.3390/mi13081346

**Published:** 2022-08-19

**Authors:** Xiaolei Bi, Lingchao Meng

**Affiliations:** 1 School of Mechanical Engineering, Henan Institute of Technology, Xinxiang 453003, China; 2 State Key Laboratory of High Performance Complex Manufacturing, Central South University, Changsha 410083, China; 3 School of Civil Aviation, Northwestern Polytechnical University, Xi’an 710072, China

**Keywords:** terahertz rectangular cavity devices, integral fabrication, uniform metallization, wire electrochemical micromachining, electrochemical deposition

## Abstract

With the development of fabrication technology for terahertz rectangular cavity devices, the fabrication process of integral terahertz waveguide cavities has received much attention because of its beneficial effect on improving the transmission of terahertz signals. However, smaller feature sizes, higher dimensional accuracy, and more stringent requirements for cavity surface roughness and edge radius make it difficult to manufacture terahertz waveguide cavities with a high operating frequency by using existing micro-manufacturing technology. At the same time, the smaller feature size also makes it more difficult to realize uniform metallization on the inner surface of a terahertz waveguide cavity. In this paper, a new and improved combined manufacturing process based on wire electrochemical micromachining and electrochemical deposition is proposed to realize the integral fabrication and uniform metallization of the inner surface of a high-frequency terahertz metal rectangular waveguide cavity. A detailed description and analysis of this combined process are carried out, together with corresponding experimental investigations. An integral 1.7 THz hollow-core metal rectangular waveguide cavity with an end-face size of 165.9 μm × 88.3 μm, an edge radius of less than 10 μm, an internal bottom surface roughness of less than 0.10 μm, and an internal side surface roughness of less than 0.40 μm was manufactured, and high-quality metallization of its inner surface was also achieved.

## 1. Introduction

A terahertz wave falls between infrared and microwave waves, which is called the gap in the electromagnetic spectrum [1]. As a prospective, pioneering, and strategic research field, terahertz technology and its applications have become the priority development field and commanding heights of science and technology at home and abroad, and its great significance to modern science and technology, national defense construction, and the national economy has been widely recognized [2]. Hitherto, terahertz technology has been applied in wireless communication, radar imaging, biomedical nondestructive testing, space detection, and many other fields and will continue to promote breakthroughs in these areas [3,4]. The generation, transmission, reception, detection, and imaging of terahertz waves require the support of various terahertz devices. The terahertz metal rectangular waveguide cavity is a typical terahertz microdevice structure, which is characterized by a rectangular hollow structure with a metal matrix in the outer layer and gold, silver, and other metal layers in the center; it is widely used, as it has the advantages of low transmission loss, good flexibility, and high security [5,6].

Various low-frequency terahertz metal rectangular cavity device structures have been fabricated through different advanced micromachining technologies. Micromachining based on deep reactive ion etching (DRIE) is one of the commonly used representative technologies [7]. Hu et al. fabricated a rectangular cavity structure for a 385 GHz bandpass filter by gold-sputtering layers onto a rectangular half-cavity with a flat surface obtained by DRIE and then performing gold–gold bonding [8]. The end-face size of the obtained rectangular cavity was 0.56 mm × 0.28 mm, the thickness of the gold layer was 5 μm, and the surface roughness was 0.5 μm. Ultraviolet-Lithographi Galvanoforming Abformung (UV-LIGA) technology based on SU-8 photoresist is also a representative technology for fabricating terahertz micro rectangular cavity structures. Shang et al. fabricated rectangular microstructures on three pieces of SU-8 photoresist with a thickness of 191 μm and then silver-plated and superimposed the surfaces [9]. Thus, a rectangular cavity structure for a WR-1.5 (Waveguide Rectangular-1.5) band third-order bandpass filter was fabricated. The measured side-wall roughness reached 45 nm, with a tolerance range of ±20 μm. Low-temperature co-fired ceramic (LTCC) technology is a cutting-edge integrated manufacturing technology that is also used for the fabrication of terahertz rectangular cavity devices. Tajima et al. used LTCC to prepare a vertical rectangular waveguide cavity and a rectangular corrugated horn cavity, and then combined them to prepare a stepped corrugated horn antenna at 300 GHz [10]. The rectangular waveguide cavity structure had a single-layer thickness of 0.2 mm and a total of 20 layers, and its end-face size was 0.8 mm × 0.4 mm. The rectangular corrugated horn structure had 27 layers with a thickness of 0.2 mm, and the size of its bottom was 0.8 mm × 0.4 mm. The rectangular cavity of each layer was prepared by drilling, and the metal layer on its surface was nickel-coated and gold-coated. In recent years, three-dimensional (3D) printing technology has been used in the manufacturing of terahertz devices [11]. Bieren et al. fabricated a terahertz linear rectangular waveguide cavity for WR-3.4 (Waveguide Rectangular-3.4) in the frequency band of 220–330 GHz by directly printing the waveguide cavity using stereo lithography appearance (SLA) technology and metallizing its inner surface by electroplating copper and sputtering gold [12]. The geometric error of the printed cavity was ±10 μm, and the thickness of the sputtered gold layer was 100 nm. Makhlouf et al. used 316 L stainless-steel particles with a size of 45 µm as materials and selective laser melting (SLM) technology to directly print a metal rectangular waveguide cavity in the WR-3 band with a frequency band of 230–320 GHz [13].

However, there are still some problems in the fabrication of high-frequency terahertz metallic rectangular waveguide cavities. This is because as the characteristic size of these devices is further reduced, the required technical indicators of the terahertz metal rectangular cavity structure, such as dimensional accuracy, surface roughness, and rounded corners, become more stringent [14]. DRIE technology has high manufacturing accuracy, but its process is complicated and prone to poor consistency of the cavity bonding position. After stacking a multi-layer rectangular cavity with UV-LIGA and LTCC, there may be problems of multi-layer alignment and a loose assembly seal. Three-dimensional printing technology realizes the integral fabrication of terahertz cavity devices, which will have a beneficial effect on the transmission of terahertz signals. For the combined manufacturing mode of SLA and surface metallization, the cavity-dimension accuracy and inner-surface roughness printed by SLA are usually poor, and it is difficult to perform uniform metallization of the inner surface of the cavity. For the direct printing mode using SLM, although the steps of surface metallization in the cavity can be reduced, it is difficult to obtain high cavity dimensional accuracy and good inner surface roughness when manufacturing terahertz cavity devices with high-operating-frequency bands using the existing technology. Therefore, manufacturing technology that can realize the high-precision manufacturing of terahertz rectangular cavity devices with a high operating-frequency band and uniform metallization of its cavity surface are highly anticipated [14].

Wire electrochemical micromachining (WECMM) uses a micro-scale metal wire as a tool cathode while controlling the motion trajectory of a wire electrode or metal workpiece through programmable software to realize the processing of micro slots, micro slots and other structures, as well as micro 3D structures with complex shapes or high aspect ratios under the conditions of specific electrolyte and electrical parameters [15]. Micro electrochemical deposition is a manufacturing technology in which metal ions are reduced to atoms under the action of electrochemistry to form metal layers on specific surfaces or metal microstructures with specific shapes by stacking layers [16,17]. Recently, a processing technology based on the combination of WECMM, electrochemical deposition, and selective dissolution has been proposed to realize the integrated manufacturing of 1 THz micro metal rectangular waveguide cavity devices. Although the overall manufacturing efficiency is reduced due to the low dissolution efficiency of the pure-nickel rectangular mandrel of WECMM, this combined manufacturing process provides a research basis for the manufacturing of terahertz metal rectangular cavity devices with high-operating-frequency bands [18].

In this paper, a new and improved combined manufacturing process based on WECMM and electrochemical deposition is proposed to manufacture high-frequency terahertz metal rectangular waveguide cavity. Taking the manufacturing process of a 1.7 THz metal rectangular waveguide cavity as an example, the process of integral manufacturing and uniform metallization of the inner surface of the waveguide cavity is described in detail.

## 2. Materials and Methods

### 2.1. Materials

In this study, a workpiece of pure aluminum (Goodfellow Ltd., Huntingdon, UK) with a thickness of 90 μm was ultrasonically cleaned before the WECMM, a 20 μm-diameter tungsten wire (Goodfellow Ltd., Huntingdon, UK) was adopted as the cathode, and the electrolytes were prepared from analytical-grade NaNO_3_ and NaCl using deionized water. The composition of the solution for gold electroplating and copper electroforming, which was obtained through repeated experimental testing [14], is listed in Table 1 and Table 2. In the selective chemical dissolution of the rectangular mandrel step, the electrolytes were prepared from analytical-grade KOH and deionized water.

### 2.2. Methods

**Figure 1:**
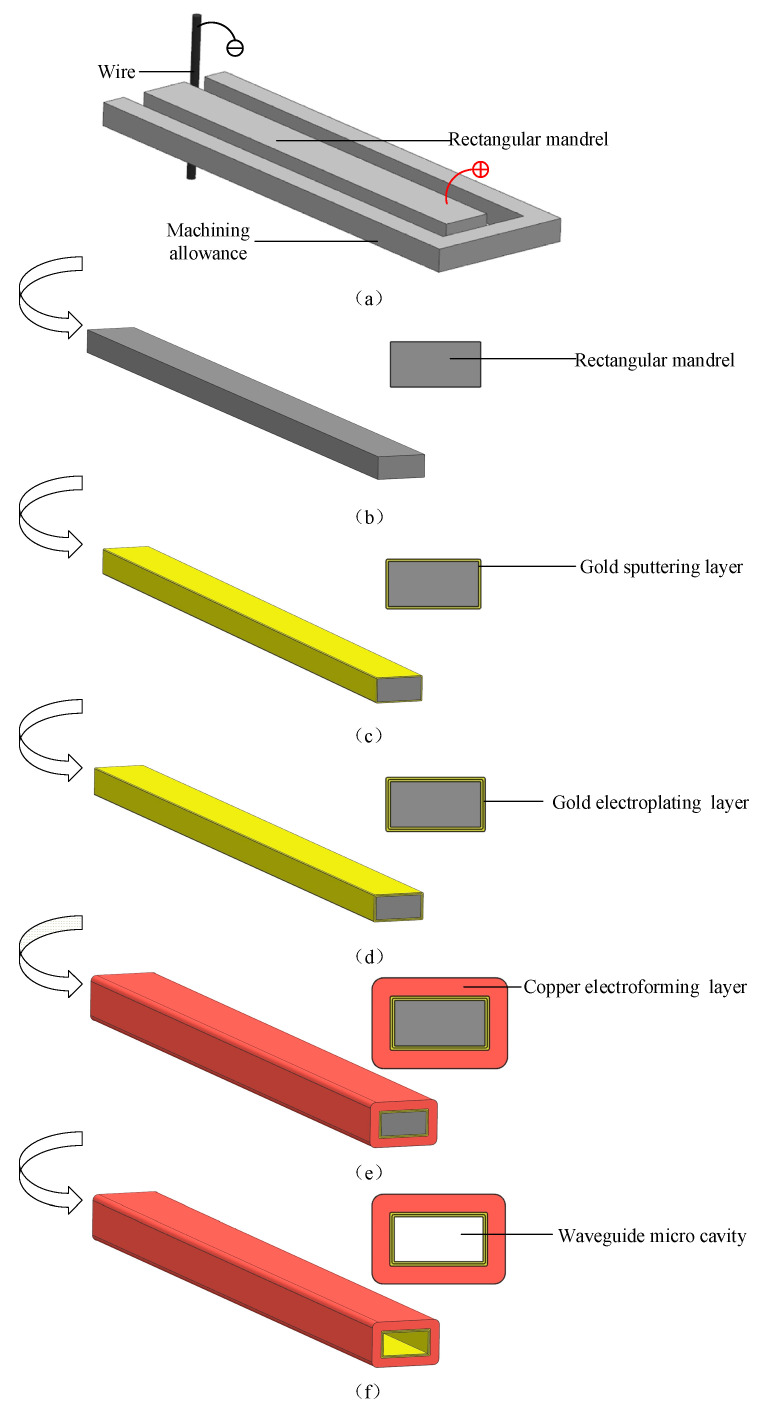
Schematic of the machining process.

The combined machining process includes WECMM, gold sputtering, gold electroplating, copper electroforming, and selective chemical dissolution, and the steps are carried out in sequence. First, a metal material that can easily be chemically dissolved by itself is processed by WECMM to obtain a rectangular mandrel, as shown in Figure 1a,b. Second, a gold layer is sputtered onto the outer surface of the rectangular mandrel, as shown in Figure 1c. Then, gold electroplating is performed on the surface of the sputtering layer, as shown in Figure 1d. After gold electroplating is completed, copper electroforming is performed on the surface of the gold electroplating layer, as shown in Figure 1e. Finally, the waveguide cavity is obtained by rapidly chemically dissolving the rectangular mandrel, as shown in Figure 1f.

According to the description of the combination process, the specific end size of the waveguide cavity will be consistent with the rectangular mandrel. Because a rectangular mandrel with an end-face size of hundreds of microns or even tens of microns can be machined through WECMM, the manufacturing of a terahertz micro-metal rectangular waveguide cavity with an end-face size of hundreds of microns or even tens of microns can be realized through this combined manufacturing process; an end-face size of this scale corresponds to that of a terahertz micro-rectangular waveguide cavity in a high-frequency band. In addition, a rectangular mandrel with good dimensional accuracy, surface roughness, and edge radius can be obtained through WECMM. The gold sputtering layer can prevent local oxidation on the surface of the rectangular mandrel, so that it is more uniform and easier to gold-electroplate. When the rectangular mandrel is rapidly chemically dissolved, a waveguide cavity with better dimensional accuracy, inner surface roughness, and edge radius will be obtained, which meets the requirements of high-frequency terahertz waveguide cavities for high-quality machining indicators.

**Figure 2:**
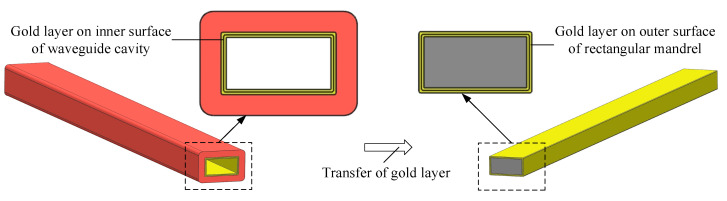
Schematic diagram of electrochemically deposited gold layer transferring from inner surface of waveguide cavity to outer surface of rectangular mandrel.

**Figure 3:**
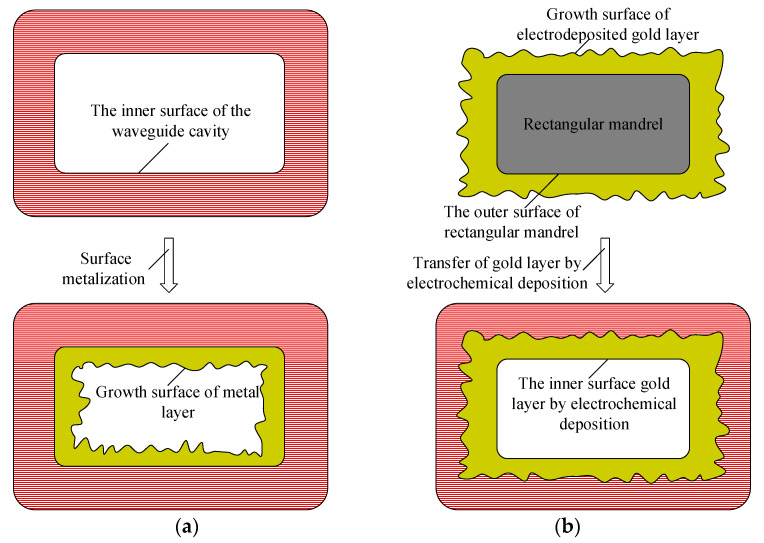
Comparison of the working surface of the metal layer inside the waveguide cavity between the original method and the presented method in this paper: (**a**) the original method, (**b**) the presented method in this paper.

In this study, the metallization of the inner surface of the waveguide cavity is achieved by electrochemical deposition transfer technology, which comprises two aspects: one is that the metallization of the inner surface of the waveguide cavity is transferred to the electrochemical deposition on the outer surface of the rectangular mandrel, and the other is that the growth surface of the metal after the metallization of the inner surface of the waveguide cavity is transferred to the non-growth surface in contact with the rectangular mandrel. The high-frequency terahertz metal rectangular waveguide cavity has a small end-face size, and it is difficult to implement metallization inside the cavity after 3D printing. The transfer of metallization inside the cavity to the outer surface of the rectangular mandrel reduces the difficulty of metallization inside the waveguide cavity with a small end-face size and improves uniformity, as shown in Figure 2. After the waveguide cavity manufactured by 3D printing is metallized, the working surface in contact with the terahertz wave inside the cavity is the growth surface of the metal layer. Due to the small size and large length-to-diameter ratio of the end-face of the waveguide cavity, it is difficult to control the quality of the growth surface of the metal layer when the surface of the waveguide cavity is metallized. In this paper, the outer surface morphology of the rectangular mandrel can be replicated by the inner surface of the gold layer. When the rectangular mandrel is chemically dissolved, the gold layer in contact with the outer surface of the rectangular mandrel will be transferred to the working surface of the gold layer inside the waveguide cavity, as shown in Figure 3.

## 3. Experimental

**Figure 4:**
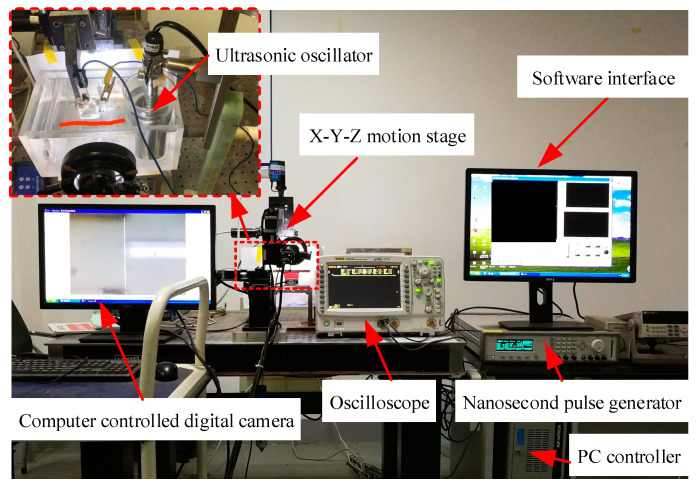
Experimental system for WECMM of rectangular mandrel.

**Figure 5:**
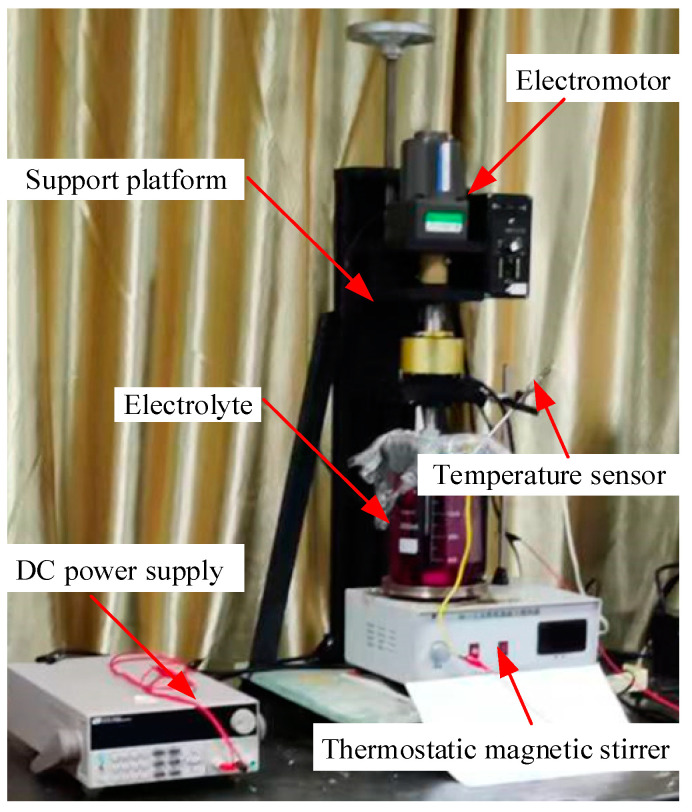
Experimental setup for the gold electroplating and copper electroforming.

The experimental system for the WECMM of the rectangular mandrel is shown in Figure 4. This system comprised an X-Y-Z motion stage, a nanosecond pulse generator (Agilent, Santa Clara, CA, USA), an oscilloscope (Tektronix, USA), a PC controller, a computer-controlled digital camera, and an ultrasonic oscillator, which could realize the function of intermittent ultrasonic vibration. The experimental setup for the gold electroplating and copper electroforming is shown in Figure 5. This system comprised a support platform, an electromotor, a direct current (DC) power supply (ITECH, China), and a thermostatic magnetic stirrer. In this study, an ultrasonic cleaner was used to perform selective chemical dissolution of the rectangular mandrel.

According to a previous study by Bi et al. [19], an optimal combination of parameters was selected for the WECMM of a pure-aluminum rectangular mandrel, as shown in Table 3. Before the experiment, the workpiece was cleaned with anhydrous ethanol and deionized water in turn, dried after cleaning, and quickly installed in the workpiece fixture and placed in an electrobath filled with electrolyte to prevent oxidation on the surface.

Referring to the work of Bi et al. [14], the vertical rotating electrochemical deposition method was used for gold plating, and the selected parameter combination is shown in Table 4. In this study, the time of gold plating was controlled at approximately 2 h. After the gold plating was completed, the workpiece was cleaned with deionized water and absolute ethanol.

Referring to the work of Bi et al. [14], the vertical rotating electrochemical deposition method was used. The selected parameter combination is shown in Table 5. The time of copper electroforming was adjusted based on 24 h.

In this study, a KOH solution with a concentration of 1 mol/L was used for chemical dissolution of the pure-aluminum rectangular mandrel. During the dissolution process, the workpiece was ultrasonically vibrated for 60 s every 0.5 h to accelerate the diffusion of the dissolved products. Every 1 h of dissolution, the workpiece was taken out to observe the end-surface topography and determine whether the aluminum rectangular mandrel was completely dissolved. After 6 h of the experiment, the pure-aluminum rectangular mandrel was completely dissolved. A 0.1 mol/L HCl solution was used to clean the workpiece for approximately 60 s to neutralize the KOH solution on the surface.

The overall and local topographies of the experimental samples were studied using a scanning electron microscope (SEM, Quanta 200, FEI, USA) and a digital microscope (DVM5000; Leica, Germany). The widths of the rectangular mandrels were measured using the digital microscope. An atomic force microscope (AFM, Dimension Edge; Bruker, Germany) was used to measure the surface roughness of the machined rectangular mandrel and the waveguide half-cavity. A measuring field of 50 μm × 50 μm was adopted in each measurement. The edge radius of the machined rectangular mandrel and the waveguide half-cavity were also measured using the same digital microscope. During the measurement of the waveguide cavity, three workpieces were randomly selected, and five positions of each workpiece were randomly selected in the direction of the end-face width and end-face height for measurement.

## 4. Results

### 4.1. Machining Process of the Integral Waveguide Cavity

**Figure 6:**
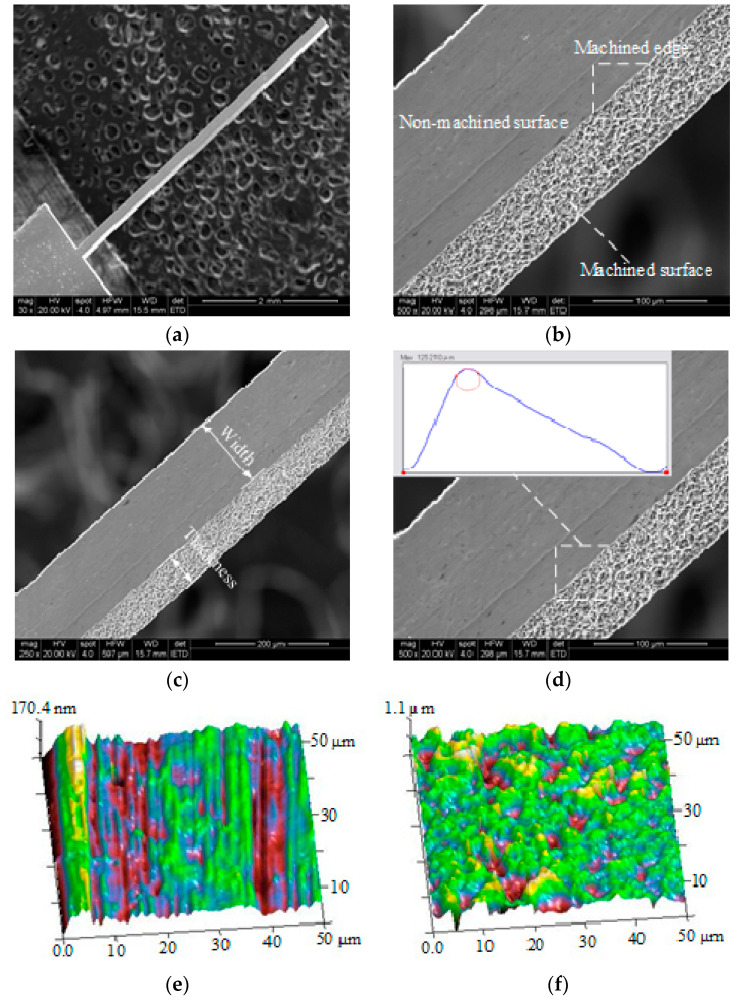
(**a**) SEM example of integral topography, (**b**) SEM image of local topography, (**c**) SEM example of the thickness and width, (**d**) SEM measurement example of the edge radius, (**e**) AFM example of the non-machined surface roughness, (**f**) AFM example of the machined surface roughness.

The end-face size of the 1.7 THz metal rectangular waveguide cavity was approximately 83 µm × 165 µm, so double-sided polished aluminum foil with a thickness of 90 µm was used to ensure the end-face thickness of the aluminum rectangular mandrel, and the end-face width of the mandrel was controlled by the tool trajectory and the side gap of WECMM. In this study, the length of the machined pure-aluminum rectangular mandrel was close to 5 mm. Morphological observations of the mandrel are shown in Figure 6, and the width and thickness, machined surface roughness, non-machined surface roughness, and edge radius of the mandrel are shown in Table 6. These data were obtained by random measurements in the length direction per millimeter. According to the measurement results in Table 6, the average end-face size of the mandrel was 86.9 µm × 165.7 µm, the average non-machined surface roughness was 0.0438 µm, the average machined surface roughness was 0.259 µm, and the average machined edge radius of pure-aluminum rectangular mandrel was 7.01 µm.

**Figure 7:**
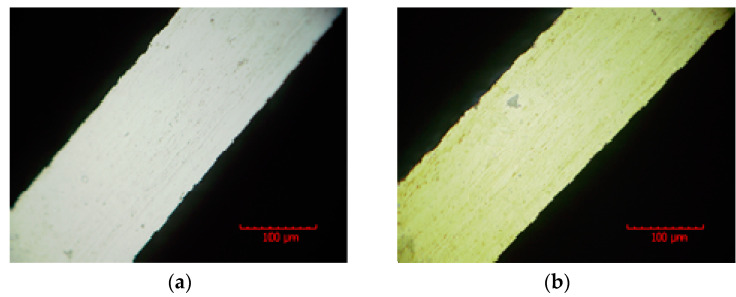
Comparison of morphology of pure aluminum rectangular mandrel before and after gold puttering: (**a**) Leica example before gold puttering, (**b**) Leica example after gold puttering.

Due to the surface oxidation of the rectangular mandrel caused by the active chemical properties of the aluminum material, the mandrel was partially unable to be electrodeposited. To solve this problem, the step of gold sputtering on the surface of an aluminum sacrificial mandrel was added before gold electroplating [20,21]. In this study, the gold sputtering was performed four times on the four surfaces of the rectangular mandrel. After the gold sputtering on each surface was completed, the gold sputtering on the next surface was performed by flipping the rectangular mandrel 90 degrees. The thickness of the gold layer was usually tens of nanometers. Morphological observations of the rectangular mandrel before and after gold sputtering are shown in Figure 7.

**Figure 8:**
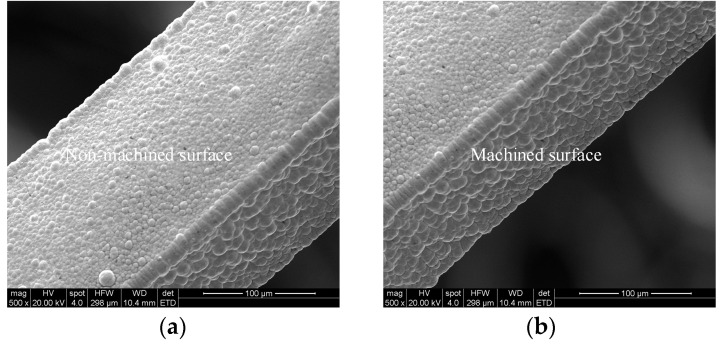
SEM examples after gold electroplating: (**a**) non-machined surface, (**b**) machined surface.

**Figure 9:**
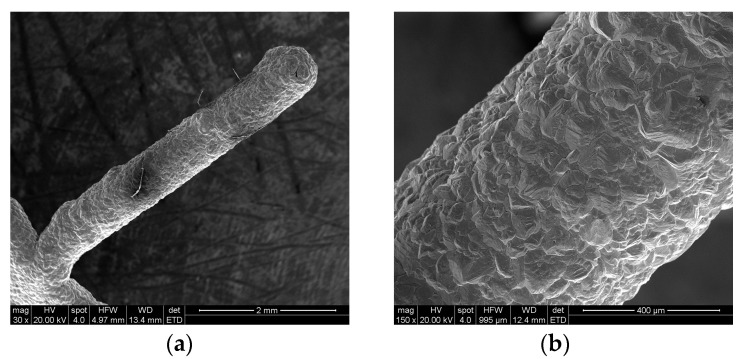
SEM examples after copper electroforming: (**a**) integral topography, (**b**) local topography.

After the gold sputtering was completed, gold electroplating was carried out on the surface of the gold sputtered layer. The morphological observation results are shown in Figure 8. The gold layer on the surface of the aluminum sacrificial mandrel was dense, and the gold layer surface was free of defects such as pores and nodules. After the gold plating, a copper electroforming experiment was carried out on the surface of the gold layer. The morphological observation results are shown in Figure 9.

**Figure 10:**
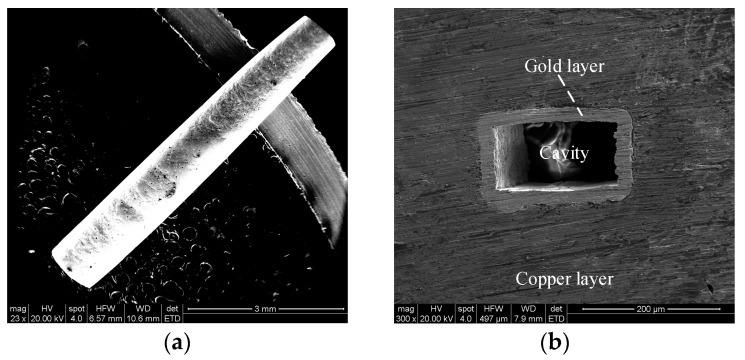
SEM examples of terahertz waveguide cavity with pure-aluminum sacrificial mandrel dissolved: (**a**) integral topography, (**b**) end-face topography.

After the copper electroforming was completed, the excess metal layer on the front end face of the workpiece was removed by grinding, and the other end of the workpiece was cut off from the workpiece matrix. The two end faces were precisely ground to expose the rectangular mandrel and obtain a better end-face morphology. The step of chemical dissolution of the pure-aluminum rectangular mandrel was carried out. The final morphological observation results are shown in Figure 10. Because the electrochemical deposition process on the outer surface of the rectangular mandrel is easy to implement and control, the gold layer transfer technology reduces the difficulty of surface metallization in the waveguide cavity compared with a traditional waveguide cavity after forming. Because a rectangular mandrel with a smaller end-face size and higher machining quality can be manufactured, the integral fabrication of a terahertz rectangular cavity device structure with a higher-operating-frequency band and uniform metallization of its cavity surface can be realized by using this proposed combined manufacturing process [18].

### 4.2. Measurement of Machining Indicators of the Waveguide Cavity

In this study, the measured machining parameters mainly include the end-face size of the waveguide cavity, the surface roughness of the waveguide cavity and the edge radius of the waveguide cavity.

**Figure 11:**
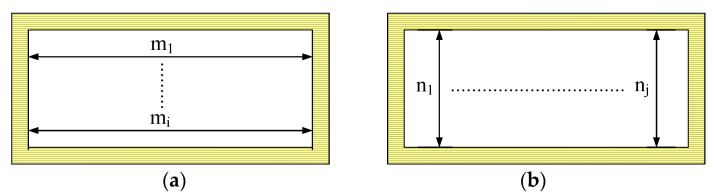
Schematic of waveguide cavity end-face dimension measurement: (**a**) end-face width, (**b**) end-face height.

**Figure 12:**
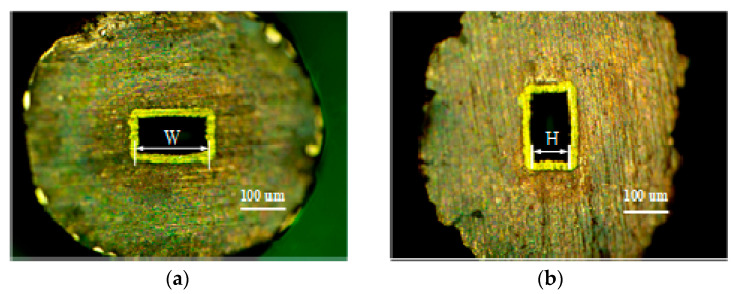
Measurement examples of waveguide cavity end-face size: (**a**) Leica example of end-face width, (**b**) Leica example of end-face height.

The measuring principle of the end-face size of the waveguide cavity is shown in Figure 11. A measurement example is shown in Figure 12. Equations (1) and (2) were used to calculate the width and height of the end face. According to the measured results, the average end width and average end height of the waveguide cavity were 165.9 μm and 88.3 μm, respectively.


(1) $$W=\frac{1}{m}\sum_{i=1}^{n}W_{i}$$

(2) $$H=\frac{1}{n}\sum_{j=1}^{n}H_{i}$$

**Figure 13:**
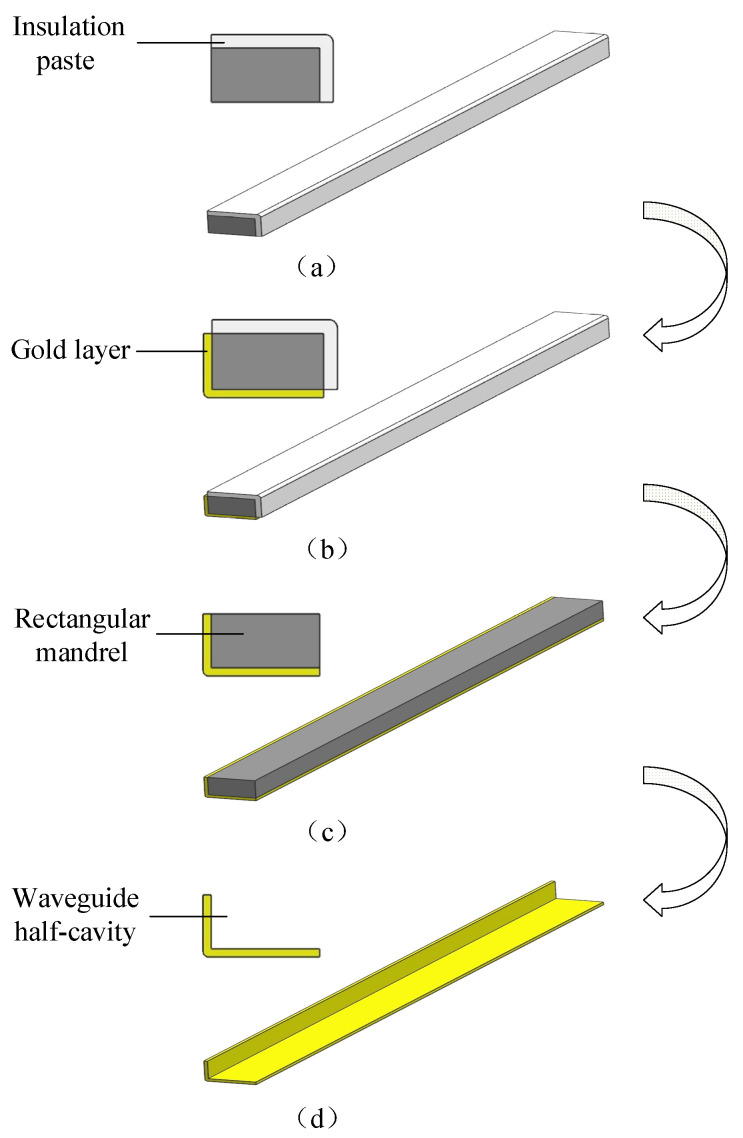
The manufacturing process of the open-type waveguide half-cavity: (**a**) local insulation of rectangular mandrel, (**b**) gold electroplating on non-insulated surface of the rectangular mandrel, (**c**) removal of the insulation paste, and (**d**) selective chemical dissolution of rectangular mandrel.

**Figure 14:**
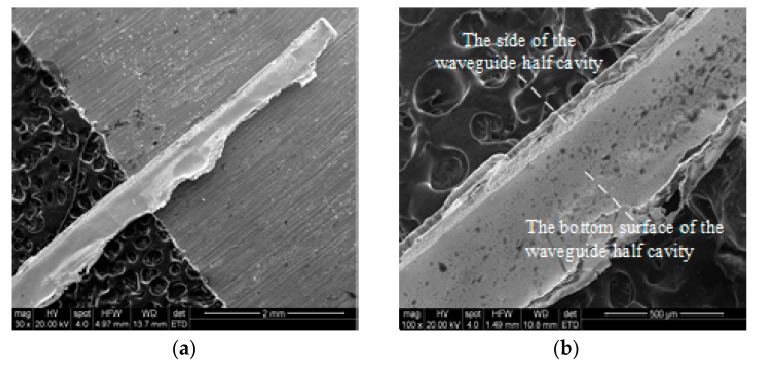
SEM examples of the open-type waveguide half-cavity: (**a**) integral topography, (**b**) local topography.

In order to measure the inner surface roughness and the edge radius of the waveguide cavity, an open-type waveguide half-cavity was prepared. The manufacturing process is shown in Figure 13 [18]. The process parameters used in the fabrication of the open-type waveguide half-cavity were consistent with those used in the fabrication of the waveguide cavity. The machined open-type waveguide half-cavity is shown in Figure 14.

**Figure 15:**
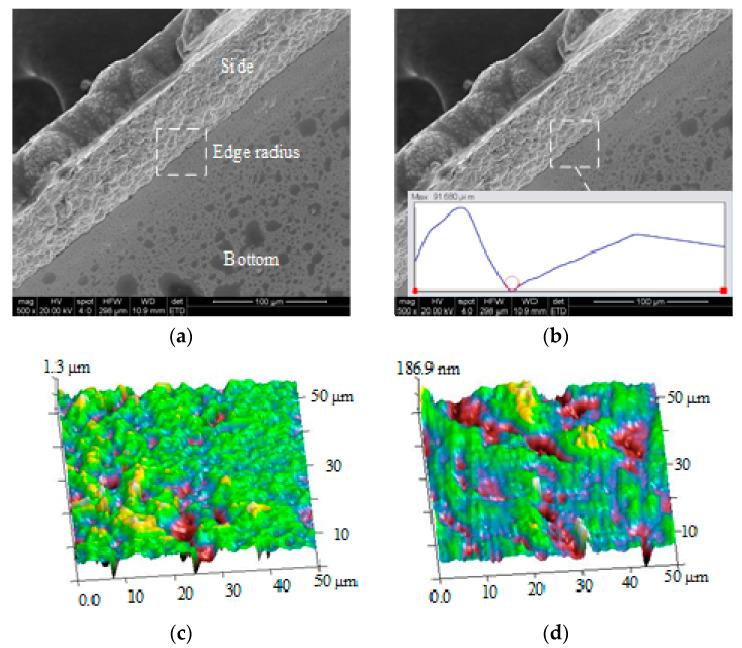
The morphology observation of the open-type waveguide half-cavity: (**a**) SEM example of local amplification morphology, (**b**) SEM example of internal edge radius, (**c**) AFM example of the inner side surface roughness, (**d**) AFM example of the inner bottom surface roughness.

In the length direction, a region per millimeter was randomly selected to measure the edge radius of the open waveguide half cavity. The final result of the edge radius was obtained by taking the average value. Similarly, a random region per millimeter in the length direction was selected to measure the internal surface roughness of the open waveguide half-cavity, and the final surface roughness result was obtained by averaging. The local amplification morphology of the open-type waveguide half-cavity is shown in Figure 15a, and the internal edge radius is shown in Figure 15b. The internal side and bottom morphology was observed via AFM, as shown in Figure 15c,d. The specific measurement results of the edge radius and surface roughness of the open waveguide cavity are shown in Table 7.

According to the measurement results, the average surface roughness of the inner side of the open-type waveguide cavity was 0.293 µm, which corresponded to the surface roughness of the rectangular mandrel of WECMM. The average surface roughness of the inner bottom of the open waveguide cavity was 0.0809 µm, which corresponded to the non-machined surface of the rectangular mandrel. The average edge radius inside the open-type waveguide cavity was 8.717 µm, which corresponded to the edge radius of the rectangular mandrel. Thus, the measurement results met the technical requirements of a 1.7 THz hollow-core metal rectangular waveguide cavity.

## 5. Conclusions

A new and improved combined manufacturing process based on WECMM and electrochemical deposition was proposed to manufacture high-frequency terahertz metal rectangular cavity devices. The following conclusions were made:

An integral 1.7 THz hollow-core metal rectangular waveguide cavity with an end-face size of 165.9 μm × 88.3 μm, an edge radius of less than 10 μm, an internal bottom surface roughness of less than 0.10 μm, and an internal side surface roughness of less than 0.40 μm was obtained. These experimental results show that this proposed manufacturing process makes it possible for the integral fabrication of terahertz rectangular cavity device structures with a high operating-frequency band.

Comparing the morphology observation results and machining measurement results of the rectangular mandrel and the waveguide cavity, the external surface morphology of the former was replicated precisely by the internal surface morphology of the latter, which indicated that the gold layer transfer technology realized the transfer of the gold layer of electrochemical deposition from the growth surface to the non-growth surface.

## Figures and Tables

**Table 1:** Composition of the solution for gold electroplating.

Component	Value
Solvent	YC-408 electroplated open-cylinder liquid
Main salt	Gold potassium citrate (3–6 g/L)

**Table 2:** Composition of the solution for copper electroforming.

Component	Value (g/L)
Copper sulfate pentahydrate	160–180
Sulfuric acid	80–100
Deionized water	

**Table 3:** The selected parameter combination for WECMM of pure aluminum rectangular mandrel.

Parameter	Value
Electrolyte	0.025 mol/L NaNO_3_ and 0.0125 mol/L NaCl
Ultrasonic oscillation frequency	40 kHz
Ultrasonic oscillation power	40 W
Ultrasonic oscillation durations	1 s
Ultrasonic oscillation intervals	30 s
Wire vibration amplitude	150 μm
Wire vibration frequency	2 Hz
Applied voltage	5 V
Feed rate	0.15 μm/s
Pulse width and period	50 ns, 5 μs

**Table 4:** The selected parameter combination for gold electroplating.

Parameters	Value
Solution temperature	55 °C
pH	3.8–4.2
Current density	0.5 A/dm^2^
Distance between anode and cathode	10 cm

**Table 5:** The selected parameter combination for copper electroforming.

Parameters	Value
Solution temperature	30 °C
Current density	0.5 A/dm^2^
Distance between anode and cathode	10 cm

**Table 6:** Measurement results of machining indicators of the pure aluminum rectangular mandrel.

Parameter	Value				
Measurement position	1	2	3	4	5
Thickness (μm)	86.9	87.1	86.5	87.3	86.8
Width (μm)	166.2	165.7	165.1	165.6	165.9
Machined surface roughness *R*_a_ (μm)	0.262	0.273	0.225	0.317	0.216
Non-machined surface roughness *R*_a_ (μm)	0.0433	0.0552	0.0376	0.0491	0.0339
Machined edge radius (μm)	6.531	7.032	7.392	6.973	7.118

**Table 7:** Measurement results of machining indicators of the open waveguide cavity.

Parameter	Value				
Measurement position	1	2	3	4	5
Side surface roughness *R*_a_ (μm)	0.238	0.311	0.272	0.305	0.339
Bottom surface roughness *R*_a_ (μm)	0.0706	0.0903	0.0819	0.0822	0.0793
Edge radius (μm)	8.093	8.971	9.023	9.165	8.334

## Data Availability

Not applicable.

## References

[1] Odintsova T.A., Koroleva A.O., Simonova A.A., Campargue A., Tretyakov M.Y. (2022). The atmospheric continuum in the “terahertz gap” region (15–700 cm^−1^): Review of experiments at SOLEIL synchrotron and modeling. J. Mol. Spectrosc..

[2] Hosako I., Sekine N., Patrashin M., Saito S., Fukunaga K., Kasai Y., Baron P., Seta T., Mendrok J., Ochiai S. (2007). At the dawn of a new era in terahertz technology. Proc. IEEE.

[3] Redo-Sanchez A., Laman N., Schulkin B., Tongue T. (2013). Review of terahertz technology readiness assessment and applications. J. Infrared Millim. Terahertz Waves.

[4] Dufour D., Marchese L., Terroux M., Oulachgar H., Généreux F., Doucet M., Mercier L., Tremblay B., Alain C., Beaupré P. (2015). Review of terahertz technology development at INO. J. Infrared Millim. Terahertz Waves.

[5] Noise L., Imaging P., Ghz W., Ampli L., Grossman E.N., Member S., Leong K., Mei X., Deal W. (2014). A Very Low Loss 220–325 GHz Silicon Micromachined Waveguide Technology. THz Lett..

[6] D’Auria M., Otter W.J., Hazell J., Gillatt B.T.W., Long-Collins C., Ridler N.M., Lucyszyn S. (2015). 3-D Printed Metal-Pipe Rectangular Waveguides. IEEE Trans. Compon. Packag. Manuf. Technol..

[7] Leong K.M.K.H., Hennig K., Zhang C., Elmadjian R.N., Zhou Z., Gorospe B.S., Chang-chien P.P., Radisic V., Member S., Deal W.R. (2012). WR1.5 Silicon Micromachined Waveguide Integration Methodology. IEEE Trans. Microw. Theory Tech..

[8] Hu J., Xie S., Zhang Y. (2012). Micromachined terahertz rectangular waveguide bandpass filter on silicon-substrate. IEEE Microw. Wirel. Compon. Lett..

[9] Shang X., Tian Y., Lancaster M.J., Member S., Singh S. (2013). A SU8 Micromachined WR-1.5 Band Waveguide Filter. IEEE Microw. Wireless. Compon. Lett..

[10] Tajima T., Song H.J., Ajito K., Yaita M., Kukutsu N. (2014). 300-GHz step-profiled corrugated horn antennas integrated in LTCC. IEEE Trans. Antennas Propag..

[11] Sun J., Hu F. (2020). Three-dimensional printing technologies for terahertz applications: A review. Int. J. RF Microw. Comput.-Aided Eng..

[12] Von Bieren A., De Rijk E., Ansermet J.P., Macor A. Monolithic metal-coated plastic components for mm-wave applications. Proceedings of the 2014 39th International Conference on Infrared, Millimeter, and Terahertz Waves (IRMMW-THz).

[13] Makhlouf S., Khani B., Lackmann J., Dulme S., Stöhr A. Metallic 3D Printed Rectangular Waveguides (WR3) for Rapid Prototyping of THz Packages. Proceedings of the 2018 First International Workshop on Mobile Terahertz Systems (IWMTS).

[14] Bi X., Meng L. (2022). Combined fabrication of terahertz hollow-core metal rectangular waveguide cavity using electrochemical deposition and selective chemical dissolution. AIP Adv..

[15] Xu K., Zeng Y., Li P., Fang X., Zhu D. (2016). Effect of wire cathode surface hydrophilia when using a travelling wire in wire electrochemical micro machining. J. Mater. Process. Technol..

[16] Chen X., Liu L., He J., Zuo F., Guo Z. (2018). Fabrication of a metal micro mold by using pulse micro electroforming. Micromachines.

[17] Wu Y., Qian S., Zhang H., Zhang Y., Cao H., Huang M. (2019). Experimental study on three-dimensional microstructure copper electroforming based on 3D printing technology. Micromachines.

[18] Bi X., Zeng Y., Dai X., Qu N. (2020). Integral fabrication of terahertz hollow-core metal rectangular waveguides with a combined process using wire electrochemical micromachining, electrochemical deposition, and selective chemical dissolution. Int. J. Adv. Manuf. Technol..

[19] Bi X., Zeng Y., Qu N. (2021). Micro-Shaping of Pure Aluminum by Intermittent Ultrasonic Oscillation Assisted Wire Electrochemical Micromachining with an Ultra-Low-Concentration Mixed Electrolyte. J. Electrochem. Soc..

[20] Ensinger W., Müller H.R. (2003). Surface treatment of aluminum oxide and tungsten carbide powders by ion beam sputter deposition. Surf. Coat. Technol..

[21] Chen Y.S., Wu C.C., Tsai J.J., Wang G.J. (2012). Electrochemical impedimetric biosensor based on a nanostructured polycarbonate substrate. Int. J. Nanomed..

